# The history of Lyme disease in Italy and its spread in the Italian territory

**DOI:** 10.3389/fphar.2023.1128142

**Published:** 2023-06-16

**Authors:** Giusto Trevisan, Maurizio Ruscio, Marina Cinco, Katiuscia Nan, Patrizia Forgione, Nicola Di Meo, Paolo Tranchini, Massimo Nacca, Silvana Trincone, Sara Giordana Rimoldi, Vania Giacomet, Michela Ricci, Davide Melandri, Stefania Artioli, Patrizia Monteforte, Giuseppe Stinco, Serena Bonin

**Affiliations:** ^1^ Department of Medical Sciences, University of Trieste, Trieste, Italy; ^2^ Azienda Sanitaria Universitaria Integrata Giuliano Isontina, and Friuli-Venezia Giulia Lyme Disease Regional Center, Trieste, Italy; ^3^ Dermatology Unit, Lyme Disease Regional Center, Naples, Italy; ^4^ Department Dermatology and Venereology, Azienda Ospedaliera di Rilevanza Nazionale Sant’Anna e San Sebastiano, Caserta, Italy; ^5^ Dermatology and Venereology Operating Unit—Bufalini Hospital, Cesena, Italy; ^6^ Microbiology, Virology, and Bioemergency Unit, Azienda Socio Territoriale Fatebenefratelli Sacco, Milano, Italy; ^7^ Department of Biomedical and Clinical Sciences L. Sacco, Milano, Italy; ^8^ Infectious Disease Unit, Internal Medicine Department S. Andrea Hospital, La Spezia, Italy; ^9^ Rheumatology Unit Genova Nervi ASL 3, Genova, Italy; ^10^ Department of Dermatology and Venereology, University of Udine, Udine, Italy

**Keywords:** Lyme borreliosis, erythema migrans, associated symptoms, antibiotic therapy, Lyme in Italy

## Abstract

Lyme borreliosis (LB) is the most common vector-borne zoonotic inflammatory disease in the Northern Hemisphere. In Italy, the first case was diagnosed in 1985 in a woman in Liguria, while the second, in 1986 in Friuli-Venezia Giulia, documenting the infection in northern Italy. Both diagnoses were confirmed by serological assessment by an indirect immunofluorescence (IFI) technique. *Borrelia* cultivation from both *Ixodes ricinus* ticks and human lesions in Trieste (Friuli-Venezia Giulia) identified *Borrelia afzelii* as the prevalent genospecies; nevertheless, *Borrelia garinii*, *Borrelia burgdorferi* (*sensu stricto*), and *Borrelia valaisiana* (VS116 Group) were also detected, although less frequently. LB was also documented in other Italian regions: in Tuscany (1991), Trentino–Alto Adige (1995–1996), Emilia-Romagna (1998), Abruzzo (1998), and more recently, Lombardy. Nevertheless, data on LB in other Italian regions, especially in southern Italy and islands, are poor. The aim of this study is to document the spread of LB in Italy through the collection of data from LB patients in eight Italian hospitals located in different Italian regions. Diagnostic criteria for LB diagnosis are as follows: i) the presence of erythema migrans (EM) or ii) a clinical picture suggestive of LB, confirmed by serological tests and/or PCR positivity for *Borrelia* detection. In addition, data also included the place of residence (town and region) and the place where patients became infected. During the observation period, 1,260 cases were gathered from the participating centers. Although different in extent from northern Italy to central/southern Italy, this study shows that LB is widespread throughout Italy.

## 1 Introduction

Lyme borreliosis (LB) is an anthropozoonotic infection transmitted by hard ticks. It is widespread mainly in the Northern Hemisphere (Trevisan et al., 2021). In Europe, LB is transmitted mainly by *Ixodes ricinus* ticks*.* Nevertheless, other vectors could be implicated in its transmission, such as *Ixodes gibbosus* in Abruzzi in Italy (Khoury et al., 1994). In Italy, the first case of LB was diagnosed in a woman in north-western Italy in Liguria in 1985 (north-western Italy) by Crovato et al. (1985), while the second case was described 1 year later in a young woman in Friuli-Venezia Giulia by Trevisan (1986), supporting the presence of this vector-borne disease even in north-eastern Italy. Both cases were confirmed by serological assessment, which was carried out in Bari by Prof. Fumarola, by an indirect immunofluorescence technique (Fumarola et al., 1985). The first study on the epidemiology of LB in Italy was presented in the “Second International Symposium on Lyme Disease and Related Disorders” in 1985 in Vienna (Trevisan et al., 1987). Further epidemiological studies were carried out, especially in Friuli-Venezia Giulia (Cinco et al., 1993) and Liguria (Cimmino et al., 1992). In Trieste, *Borrelia* (Friuli-Venezia Giulia) culture in the BSK medium was also carried out, both from *Ixodes* ticks (Cinco et al., 1989) and patients’ biopsies, namely, erythema migrans (EM) (Cinco et al., 1992) and annular/roseolar erythema (Trevisan et al., 1992), and from the human myocardium (Lardieri et al., 1993). The isolation and identification of Borreliae from ticks identified *Borrelia afzelii* as the most widespread genospecies in Friuli-Venezia Giulia, where *Borrelia garinii*, *Borrelia burgdorferi* (*sensu stricto*) (Ciceroni et al., 2001), and *Borrelia valaisiana* (VS116 Group) could also be found (Cinco et al., 1998).

The Regional Centre (Friuli-Venezia Giulia) of reference for Lyme disease (resolution of the Regional Council No. 1956/1993) appointed by the Ministry of Health (Note No. 1400.2/26.N/2445 of 9 April 1997) was established at the Dermatology Clinic in Trieste on 22 April 1993 as a supra-regional center. Patients from all over Italy have come there, gathering information on the spread of LB throughout Italy.

In 1991, Lyme Borreliae were detected for the first time in Veneto (Trevisan et al., 1991), where they were subsequently isolated from more than 50 patients (Ciceroni et al., 2001). Since that time LB has been endemic in that region (Beltrame et al., 2021).

Certain cases of LB have also been reported in Tuscany (Stefanelli et al., 1994), with the following identification of *Borrelia lusitaniae* (Bertolotti et al., 2006), and in Trentino (Merler et al., 1996) and Alto Adige (South Tirol) (Cacciapuoti et al., 1995), where *B. burgdorferi* (*sensu stricto*), *B. garinii*, and *Borrelia* group VS461 were identified. Additional endemic areas in Italy were notified in Emilia-Romagna (Gaddoni et al., 1998), Abruzzi (Fazii et al., 2000), and Lombardy (Rimoldi et al., 2020). Data from southern Italy and islands are limited (Zanet et al., 2020). Sporadic cases have been reported in Sicily (Rinaldi et al., 1991), Sardinia (Ruata et al., 1992), Lazio (Frediani et al., 1993), and Calabria (Santino et al., 1996).

Over the past 15 years, LB cases have considerably increased in endemic regions of Europe and have emerged in new geographic areas (Stark et al., 2023). In the United States, Lyme disease is highly endemic in the Northeast, Middle Atlantic, and Upper Midwest regions, and the incidence is increasing in neighboring states (Schwartz et al., 2017). In Europe, the highest incidence of LB is in Estonia, Slovenia, Switzerland, Holland (Stark et al., 2023), and Norway (Eliassen et al., 2017) with more than 100 cases/100,000 inhabitants/year. In Lithuania, the incidence is 99.9/100,000 inhabitants per year (Petrulioniene et al., 2020), in Finland 99.6, and in Germany and Poland 37. However, the highest rate is in Sweden, in Blekinge, with 632/100,000 inhabitants/year (Vandekerckhove et al., 2021).

The aim of the present study is to document the spread of LB in Italy through the collection of data from LB patients in eight Italian hospitals located in different Italian regions.

## 2 Patients and methods

**FIGURE 1:**
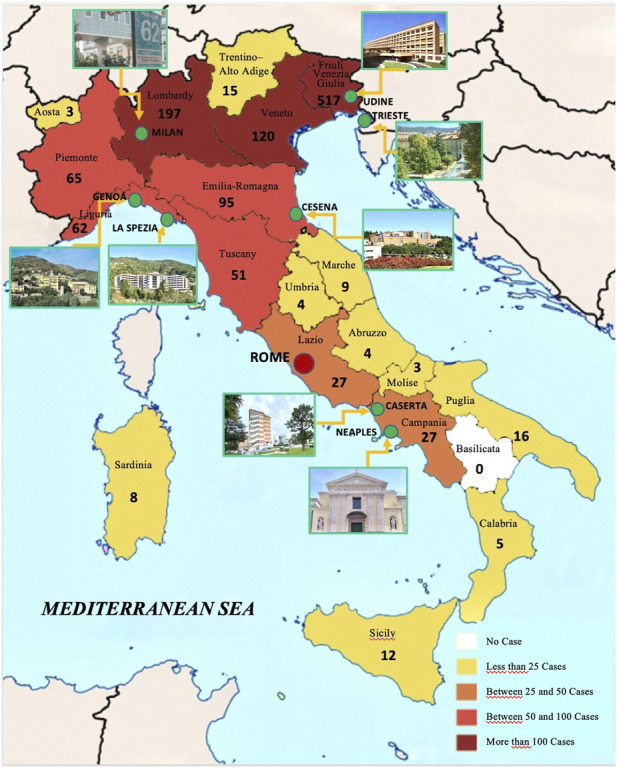
Map of Italy with participating centers and number of cases detected in each region during the observation period.

Patients’ data were gathered from 01/01/2010 to 30/08/2022 in the participating centers in Friuli-Venezia Giulia, Liguria, Lombardy, Emilia-Romagna, and Campania, as shown in Figure 1. During the COVID pandemic, telemedicine has also been used (Trevisan et al., 2022) for diagnosis, especially for EM lesions. In each center, the following diagnostic criteria were applied for LB diagnosis:

1. The presence of an EM lesion that is pathognomonic for LB
2. A clinical picture suggestive of LB, confirmed by two-titer serological tests and/or by direct detection methods such as PCR and *Borrelia* culture. In addition to EM, symptoms suggestive of LB were conjunctivitis, migratory arthralgia, myalgia, arrhythmia, headache, involvement of cranial nerves, and poly-meningo-radicolo-neuritis, when appearing after the tick bite.

For each patient, the following data were gathered: age at diagnosis, gender, the year of diagnosis, the Italian region of residence, the geographical area of infection if different from residence, the recollection of a tick bite, the anatomical site of EM occurrence, other symptoms, and the antibiotic treatment. The following associated symptoms were recorded: fever, lymphadenopathy, migratory arthralgias, arthritis; at the skin level (in addition to EM), annular multiple erythema, *Borrelia* lymphocytoma, primary cutaneous B-cell marginal zone lymphoma, and acrodermatitis chronica atrophicans; at the muscular system, asthenia and myositic symptoms; at the nervous system, headache, meningo-encephalitis, facial palsy and other cranial nerves, poly-meningo-radiculus-neuritis (Miele et al., 2022), paresthesia, cognitive disorders, anxiety, and depression; at the cardiac level, rhythm disorders, myocarditis, pericarditis, and POT’s syndrome; at the ocular level, conjunctivitis, optic neuritis, uveitis, and neuroretinitis; dizziness; and Jarisch–Herxeimer reaction.

According to the residence of patients, cases were grouped in the following categories:

1- Northern Italy for patients living in Valle d'Aosta, Piemonte, Lombardy, Trentino-Alto Adige, Veneto, Friuli-Venezia Giulia, Liguria, and Emilia-Romagna.
2- Central Italy for patients living in Abruzzo, Lazio, Marche, Tuscany, and Umbria.
3- Southern Italy and islands for patients living in Apulia, Basilicata, Calabria, Campania, Molise, Sardinia, and Sicily.

### 2.1 Indirect methods for Lyme *Borrelia* detection

Detection of antibodies against the *B. burgdorferi* sensu lato complex was performed by conventional two-tiered serologic testing, according to European and North American guidelines (Eldin et al., 2019). The first step was the ELISA test or the chemiluminescence test (CLIA), which were confirmed in the case of positivity or doubtful results by immunoblot. Serological tests were carried out in all patients except those with manifest EM. ELISA, CLIA, and immunoblots were provided by EUROIMMUN.

### 2.2 PCR analysis

PCR analysis was carried out in DNA obtained by tissue biopsies or synovial fluid or blood amplifying two *Borrelia* targets, namely, a fragment of OspA and flagellin gene, as already reported (Bonin et al., 2016; di Meo et al., 2015; Ivacic et al., 2007; Jenkins et al., 2012; Pauluzzi et al., 2004).

### 2.3 Statistical analyses

Data were gathered in a database and submitted to statistical analyses. A descriptive analysis for each individual variable considered in this study was carried out. Continuous variables were described with a mean value and standard deviation. Associations were tested by the chi-squared test or the Fisher exact test. A *p*-value less than 0.05 was considered significant. Statistical analysis was carried out using the Stata/SE 16.0 package (StataCorp, College Station, TX, United States).

## 3 Results

During the observation period, 1,260 patients were diagnosed with LB in Italy in the clinical centers participating in the study. Cases were grouped according to the region of residence, as shown in Figure 1; Table 1. The overall distribution of patients per gender did not differ across Italy, as reported in Table 1 (*p* = 0.6).

**TABLE 1:** Patients with LB per geographical area and genders.

Geographical area	Male	Female	Total
Northern Italy	500 (40%)	593 (47%)	1,093 (87%)
Central Italy	41 (3%)	54 (4%)	96 (7%)
Southern Italy with islands	29 (2%)	42 (3%)	71 (5%)
Total	570 (45%)	690 (55%)	1,260 (100%)

**FIGURE 2:**
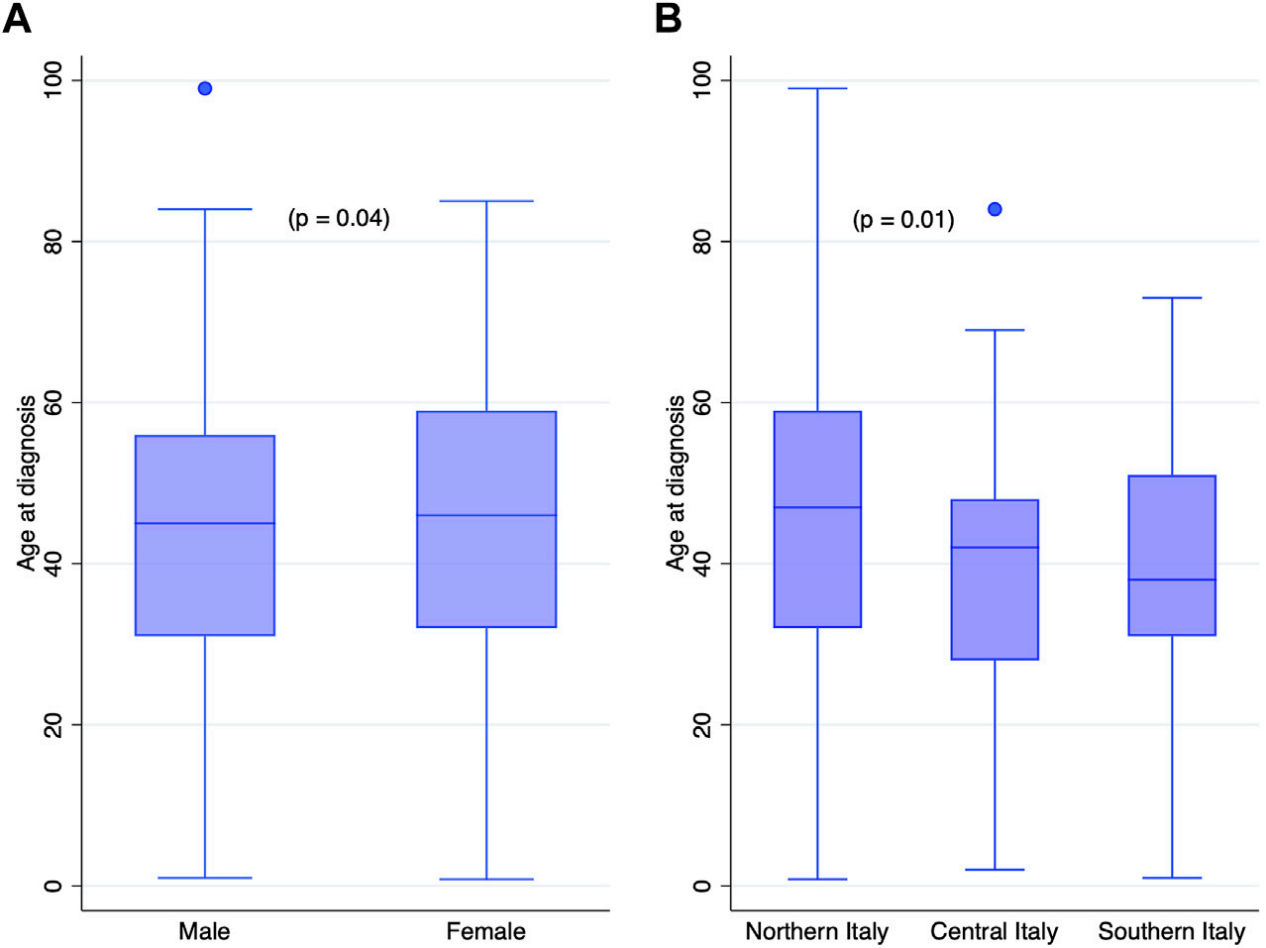
Age at diagnosis by **(A)** genders and **(B)** geographical area of residence.

The mean age at diagnosis was 43 years (S.D. 20.0). Age at diagnosis varied slightly per gender with a higher age at diagnosis for women (44 years, 95% CI: 43.0–46.0) vs. men (42 years, 95% CI: 40.5–43.9) (*p* = 0.04), as shown in Figure 2A. Age at diagnosis also varied with the geographical area of residence (*p* = 0.01); notably, patients living in northern Italian regions were significantly older than those living in central Italy. In addition, a decreasing trend in age from northern to southern Italy was observed (*p* = 0.001). Accordingly, the mean age of patients from northern Italy was 44 years (95% CI: 42.8–45.3), from central Italy was 38 years (95% CI: 34.5–42.1), and from southern Italy and islands was 41 years (95% CI: 37.0–44.3).

A trend (*p* = 0.001) of growing cases across the years of observation was recorded in all geographical areas. A maximum of cases was observed during 2020 with 211 diagnoses.

Regarding the tick bite, 936 of the 1,260 patients recalled a tick bite (74%), while 324 did not do so (26%), with any difference between genders (*p* = 0.4). On the contrary, the recollection of the tick bite was associated with the geographical area of residence (*p* = 0.02) with a significantly higher number of patients recalling a tick bite in southern Italy and islands and a considerably lower number in central Italy. The development of EM was also associated with the recollection of a tick bite (*p* < 0.001); notably, 86% of patients recalling a tick bite developed EM. Anyway, the development of EM did not differ with the geographical area (*p* = 0.3) and genders (*p* = 0.2).

The body sites where EM developed were grouped in the i) head and neck; ii) trunk; iii) higher limbs; and iv) lower limbs. Of the entire cohort, 943 (75%) patients had an EM, as shown in Table 2. In 2% of cases, the location of the EM was not included in clinical records. As shown in Table 2, the lower limbs were the preferential site for EM, with 54% of EM in this site. The trunk was the second most common site, accounting for 25% of the total cases. Anyway, the distribution of the anatomical sites of EM did not differ between genders (*p* = 0.8).

**TABLE 2:** Anatomical site of EM in pediatric (aged <14 years) and adult patients.

Anatomical site	Patients aged <14 years			Patients aged ≥14 years			All patients		
	Male	Female	Total	Male	Female	Total	Male	Female	Total
Head and neck	20 (17%)	16 (13%)	36 (30%)	14 (1.5%)	21 (2.5%)	35 (4%)	34 (3%)	37 (4%)	71 (7%)
Trunk	23 (19%)	15 (13%)	38 (32%)	90 (11%)	105 (13%)	195 (24%)	113 (12%)	120 (13%)	233 (25%)
Higher limbs	8 (7%)	3 (2%)	11 (9%)	37 (4%)	56 (7%)	93 (11%)	45 (5%)	59 (6%)	104 (11%)
Lower limbs	18 (15%)	15 (13%)	33 (28%)	214 (26%)	265 (32%)	479 (58%)	232 (25%)	280 (29%)	512 (54%)
Unknown	0 (0%)	2 (1%)	2 (1%)	12 (2%)	9 (1%)	21 (3%)	12 (1%)	11 (1%)	23 (2%)

In 120 patients, EM developed in pediatric patients (aged <14 years). In those patients, the most frequent site for EM was the trunk (32% of EM), followed by the head and neck (30% of pediatric EM) and lower limbs (28% of EM in children), but none of them prevailed, as shown in Table 2. As for the overall cohort, the location of EM did not differ between genders (*p* = 0.4).

Taken those observations, patients’ age varied significantly with the EM location (*p* < 0.0001), with the lowest age for the head and neck (26 years) and the highest for lower limbs (46 years).

### 3.1 Symptoms and clinical features

LB manifested only with EM in 380 patients (30%), while in 563 patients (45%), other symptoms occurred in addition to EM. The presence of associated symptoms in patients with EM differed significantly with respect to genders; female patients had, indeed, a higher rate of associated symptoms than males (*p* = 0.02), Table 3. Furthermore, patients with EM rash at the head and neck had a higher rate of associated symptoms (79%, *p* < 0.001), even considering only adult patients (*p* < 0.001) with 90% of patients with EM at the head and neck developing associated symptoms.

**TABLE 3:** List of recorded symptoms per genders.

Patients with EM (N = 943)	Male	Female	Total
W/o associated symptoms	193 (20%)	187 (20%)	380 (40%)
With associated symptoms	243 (26%)	320 (34%)	563 (60%)
Associated symptoms in patients with EM^a^			
Articular symptoms	106 (12%)	148 (17%)	254 (29%)
Neurological symptoms	96 (11%)	136 (15%)	232 (26%)
Muscular symptoms	88 (10%)	127 (14%)	215 (24%)
Skin (excluded EM)	50 (6%)	98 (11%)	148 (17%)
Fever	61 (7%)	69 (8%)	130 (15%)
Ocular symptoms	22 (2%)	32 (4%)	54 (6%)
Cardiac symptoms	17 (2%)	26 (3%)	43 (5%)
Associated symptoms in patients w/o EM^a^	134 (15%)	183 (21%)	317 (36%)
Articular symptoms	92 (10%)	125 (14%)	217 (24%)
Neurological symptoms	66 (8%)	118 (13%)	184 (21%)
Muscular symptoms	74 (8%)	101 (11%)	175 (19%)
Fever	23 (3%)	45 (5%)	68 (8%)
Skin (excluded EM)	13 (1%)	36 (4%)	49 (5%)
Ocular symptoms	15 (2%)	24 (3%)	39 (5%)
Cardiac symptoms	13 (1%)	22 (3%)	35 (4%)

^a^ Percentage values are calculated, considering only the group of patients with associated symptoms (N = 880).

**FIGURE 3:**
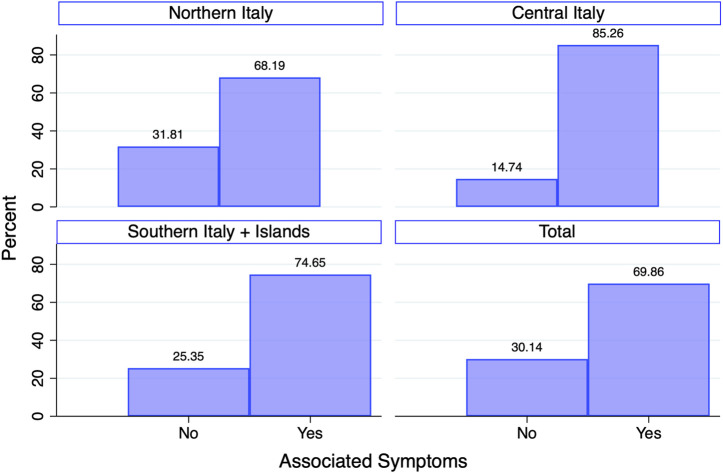
Distribution of patients according to the presence of associated symptoms per geographical areas, namely, northern, central, and southern Italy with islands.

Patients without EM, with other symptoms suggestive of LB, were 317 (25%). Considering the place of residence, the presence of associated symptoms was higher in patients from central Italy (85%) than that in northern Italy (68%) (*p* = 0.002), as depicted in Figure 3. In northern Italy, 348 (32%) patients had EM without associated symptoms, while they dropped down to 15% (14 out of 96) in central Italy and 25% (18 out of 71) in southern Italy (*p* = 0.001).

Overall, in 880 patients (70%), associated symptoms occurred as reported in Table 3.

In patients with associated symptoms, skin lesions other than EM were significantly higher in patients with EM (*p* < 0.001), while articular (*p* < 0.001), muscular (*p* < 0.001), and neurological (*p* < 0.001) symptoms were significantly higher in patients without EM. On the contrary, fever (*p* = 0.7), ocular (*p* = 0.4), and cardiac symptoms (*p* = 0.07) did not seem to be associated with the development of EM.

### 3.2 Serological data

Serological tests were carried out in 947 patients (75% of the entire cohort). The rate of positive serology (both IgG or IgM) did not vary with gender (*p* = 0.3) and with the patients’ residence (*p* = 0.1). Serological tests were performed in 687 out of 943 patients with EM (73%) and in 303 out of 313 (96%) patients without EM. Since EM is pathognomonic for LB, there is no need to perform serological tests when EM is diagnosed (Schriefer, 2015). This is the main reason why serological tests were not performed in a certain number of patients with EM rashes. The results on serology are reported in Table 4.

**TABLE 4:** Serological data.

	All patients (%)	Patients with EM = 943 (%)	Patients w/o EM = 317 (%)
Cases w/o serology	273/1,260 (22%)	259/943 (27%)	14/317 (4%)
Cases with serology	987/1,260 (78%)	684/943 (73%)	303/317 (96%)
Positive IgM or IgG	872/987 (88%)	597/684 (87%)	275/303 (91%)
Negative	115/987 (12%)	87/684 (13%)	28/303 (9%)

In patients with EM, the duration of the disease varied significantly concerning serological results (*p* = 0.02) with a mean value of 2 months in those with negative serological tests. The same test in patients without EM did not show any significant result (*p* = 0.7).

### 3.3 Molecular analysis in patients with negative serology

In 14 patients (12 women and 2 men) without EM and negative serological results, LB diagnosis was based on a positive *Borrelia* detection by PCR. In seven cases, PCR was carried out in DNA from blood, in one from eye swab, in two cases from CSF, and in four from skin biopsies, namely, two primary cutaneous B-cell marginal zone lymphoma (PCMZL) (Gatti et al., 2014), one roseolar erythema, and one interstitial granulomatous dermatitis (IGD) (di Meo et al., 2015).

### 3.4 Therapy

The therapeutical regimen was based on the use of the following antibiotics given per os: amoxicillin (1 g 3 times die, 14–21 days); POM penicillin (1*10^6^ UI 3 times die, 14–21–28 days); doxycycline (100 mg 2 times die, 14–21–28 days); minocycline (100 mg 2 times die, 14–21-28 days); cefuroxime (500 mg 2 times die, 14–21–28 days); azithromycin (500 mg 1 die, 7–9 days); clarithromycin (500 mg 2 times die, 14–21–28 days). Ceftriaxone (2–3 g 1 die, 14–21–28 days) and penicillin G (5–6*10^6^ UI 4 times a die, 14 days) were given intravenously. The distribution of the therapeutical regimen differed in patients with EM from those without EM, as shown in Table 5 (*p* < 0.001). EM patients were mostly treated with amoxicillin and doxycycline, while those without EM had a higher rate of intravenous therapy.

**TABLE 5:** Therapeutical regimen in patients with EM and w/o EM.

Antibiotic	Erythema migrans	W/o erythema migrans	Total
Per os			
Amoxicillin	466 (37%)	80 (6%)	546 (43%)
POM penicillin	4 (0.3%)	2 (0.2%)	6 (0.5%)
Doxycycline	336 (27%)	97 (7.5%)	433 (34%)
Minocycline	20 (2%)	15 (1%)	35 (3%)
Cefuroxime	8 (0.6%)	4 (0.3%)	12 (1%)
Azithromycin	47 (3.7%)	10 (0.8%)	57 (4.5%)
Clarithromycin	16 (1.3%)	4 (0.3%)	20 (2%)
Intravenously			
Ceftriaxone	45 (3%)	96 (8%)	141 (11%)
Penicillin G	1 (0.1%)	9 (0.7%)	10 (1%)
Total	943 (75%)	317 (25%)	1,260 (100%)

The therapeutical regimen differed concerning the geographic area of residence, as shown in Table 6 (*p* < 0.001). This is mostly due to the occurrence of associated symptoms that varied significantly with the geographic area of residence. Nevertheless, in early Lyme disease, amoxicillin was usually preferred in northern Italy, while doxycycline, in central and southern Italy. This difference was maintained even by the exclusion of pediatric patients for whom doxycycline is not recommended. In northern Italy, amoxicillin (40%) continued to prevail, while doxycycline, in central (42%) and southern Italy (28%). Data on adult patients also showed the highest rate in the use of intravenous therapy in central and southern Italy as a sign of late Lyme disease treatment.

**TABLE 6:** Therapeutical regimen per geographical area in the overall cohort.

Antibiotic	Northern Italy	Central Italy	Southern Italy + island	Total
Per os				
Amoxicillin	493 (45%)	34 (35.5%)	19 (27%)	546 (43%)
POM penicillin	6 (1%)	0 (0%)	0 (0%)	6 (0.5%)
Doxycycline	378 (34%)	35 (36.5%)	20 (28%)	433 (34%)
Minocycline	29 (3%)	3 (3%)	3 (4%)	35 (3%)
Cefuroxime	10 (1%)	0 (0%)	2 (3%)	12 (1%)
Azithromycin	47 (4%)	5 (5%)	5 (7%)	57 (4.5%)
Clarithromycin	17 (2%)	1 (1%)	2 (3%)	20 (2%)
Intravenously				
Ceftriaxone	103 (9%)	18 (19%)	20 (28%)	141 (11%)
Penicillin G	10 (1%)	0 (0%)	0 (0%)	10 (1%)
Total	1,093 (100%)	96 (100%)	71 (100%)	1,260 (100%)

## 4 Discussion

This study is based on the collection of data from LB patients in different clinical centers for Lyme disease in Italy to obtain a picture of the disease throughout Italy. The centers involved in the study are located in northern Italy (6 centers: Friuli-Venezia Giulia, 2; Lombardy and Liguria, 2; Emilia-Romagna, 1) and southern Italy (Campania, 2) to cover the entire Italian territory. The observation period spanned between 2010 and 2022 to harmonize the collection of data since in southern Italy, in previous years, there was no particular surveillance on LB. In Europe, the most common tick-borne disease is LB (Marques et al., 2021). Its incidence is increasing in different Western European countries together with a geographical expansion of the disease into previously non-endemic areas (Beltrame et al., 2021). Although we are not describing the incidence of the disease, cases increased in the observation period with a maximum of observations in 2020, possibly due to the COVID pandemic and the use of telemedicine in diagnosing EM (Trevisan et al., 2022). In Italy, during the pandemic, patients could not reach hospitals for dermatological evaluation, and after a telephone interview with the doctors, they sent their pictures of the lesions by e-mail or showed them on a video call.

Most patients with LB in our cohort came from northern Italy, where LB has been reported with higher frequencies (Rimoldi et al., 2020; Beltrame et al., 2021). Nevertheless, certain cases were also diagnosed in central and southern Italy in agreement with the presence of *Ixodes* vectors and Borreliae in those areas (Stefanelli et al., 1994). Although the number of cases in central and southern Italy was significantly lower, our study highlights the presence of Lyme group *Borrelia* even there. Patients who became infected in different regions, where they lived, were very limited in our study (39), and 17 of them lived in southern Italy. This means that 24% of LB patients diagnosed in southern Italy in our cohort were infected in other countries (9) or in different Italian regions, but the residual 76% became infected in their region, documenting the presence of Lyme group Borreliae. Although not in humans, seroprevalence of *B. burgdorferi* in stray dogs, as sentinel animals for tick-borne infection, has been described in Sicily (Galluzzo et al., 2020) and *B. burgdorferi* sensu stricto and *B. afzelii* were identified in *Ixodes* ticks in southern Italy and islands (Zanet et al., 2020) and in central Italy (Pascucci and Cammà, 2010; Mancini et al., 2019) in agreement with our findings. Regarding the clinical manifestations, 85% of patients from central Italy had LB with associated symptoms documenting LB at least in an early disseminated stage. It is, indeed, well recognized that in non-endemic areas, LB diagnosis could be elusive (Maxwell, 2020) with the possible consequence of the dissemination of the disease from a local stage to an early or late disseminated phase.

Another significant geography-related difference in this study is the therapeutic regimen: in northern Italy, most patients were treated with amoxicillin, while in central and southern Italy, with doxycycline, and this was confirmed even by the exclusion of pediatric patients for whom doxycycline is not recommended (Wormser et al., 2019). The difference in the antibiotic regimen is possibly related to the diffusion in central and southern Italy of the *Rickettsia* species (Scaffidi, 1981; Selmi et al., 2017), for which the treatment of choice is doxycycline (Spernovasilis et al., 2021). Furthermore, patients treated intravenously with penicillin G or ceftriaxone were considerably higher in central (19%) and southern Italy (28%) when compared to northern Italy (10%), supporting for a diagnosis of late LB. Patients presenting with EM as the only LB symptom prevailed, indeed, in northern Italy. Therefore, difficulties in the diagnosis in non-endemic areas together with the lack of information on LB among inhabitants could explain those results. On the contrary, the recollection of the tick bite was higher in inhabitants of southern Italy and islands. Patients also differed for age at diagnosis, with older patients in northern Italy than central and southern Italy, which is in line with the differences in age among Italian region inhabitants (http://www.comuni-italiani.it/statistiche/eta.html, accessed 16/12/2022 2022). The mean age in northern Italy is, indeed, reported to be higher than that in southern Italy, explaining our findings. The body location of EM rashes confirmed previous reports with the prevailing site in adults being the lower limbs, followed by the trunk (Rebman et al., 2021). Anyway, in pediatric patients, EM rashes were differently distributed with similar occurrences in the head and neck, trunk, and lower limbs, as already found (Backman and Skogman, 2018).

EM rashes at the head and neck resulted in a higher risk of developing additional symptoms in LB in our cohort in agreement with other reports, both in children (Backman and Skogman, 2018) and adults (Ogrinc et al., 2022).

Overall, the collection of data from eight clinical centers in different geographic areas in Italy documented the presence of LB even in southern Italy and islands, although with considerably lower rates than northern Italy, where LB is endemic in certain regions. Nevertheless, we acknowledge that the main limitation to this study is the incomplete information underestimating LB in Italy, especially for certain regions where LB is endemic (i.e., Trentino-Alto Adige, Piemonte, and Valle d’Aosta).

## Data Availability

The raw data supporting the conclusion of this article will be made available by the authors, without undue reservation.
